# SENSE-Cog Asia: A Feasibility Study of a Hearing Intervention to Improve Outcomes in People With Dementia

**DOI:** 10.3389/fneur.2021.654143

**Published:** 2021-06-14

**Authors:** Saima Sheikh, Sehrish Tofique, Nosheen Zehra, Rabia Amjad, Maham Rasheed, Maria Usman, Shanker Lal, Emma Hooper, Jahanara Miah, Nusrat Husain, Hussain Jafri, Nasim Chaudhry, Iracema Leroi

**Affiliations:** ^1^Division of Neuroscience and Experimental Psychology, University of Manchester, Manchester, United Kingdom; ^2^Department of Psychiatry, Pakistan Institute of Living and Learning, Karachi, Pakistan; ^3^Department of Health, Institute of Health, University of Cumbria, Lancaster, United Kingdom; ^4^Division of Global Mental Health, University of Manchester, Manchester, United Kingdom; ^5^Department of Health, Alzheimer Pakistan, Lahore, Pakistan; ^6^Department of Health, Fatima Jinnah Medical University, Lahore, Pakistan; ^7^Department of Psychiatry, Global Brain Health Institute, Trinity College Dublin, Dublin, Ireland

**Keywords:** dementia, LMICs, hearing impairment, feasibility, acceptability, tolerability

## Abstract

**Background:** There are few evidence-based non-pharmacological interventions adapted for people with dementia (PwD) in lower- and middle-income countries (LMIC). Thus, there is value in culturally adapting existing interventions from other settings. One such intervention for PwD involves hearing rehabilitation, which may improve dementia-related outcomes.

**Objective:** To culturally adapt and evaluate the feasibility and acceptability of a multi-faceted hearing support intervention to enhance quality of life in PwD for a LMIC setting, Pakistan.

**Design:** This was a study in three phases: (1) training and capacity building to deliver the study, including Patient and Public Involvement (PPI); (2) cultural adaptation of the intervention; and (3) delivery of a single-group feasibility study with a pre-test post-test design.

**Setting:** Home-based intervention, in two cities of Pakistan.

**Participants:** Adults aged ≥ 60 with mild-moderate dementia and uncorrected or partially corrected hearing impairment, and their study partners (*n* = 14).

**Intervention:** An adapted hearing support intervention (HSI) comprising a full assessment of hearing function, fitting of hearing aids, and home-based support from a “hearing support practitioner.”

**Outcomes:** Ratings of the feasibility of the study procedures, and acceptability/tolerability of the adapted intervention were ascertained through questionnaires, participant diaries, therapist logbooks and semi-structured interviews. A signal of effectiveness of the intervention was also explored using a battery of dementia-related outcome measures.

**Results:** Following cultural adaptation and capacity building for study conduct and delivery, we successfully implemented all intervention components in most participants, which were well-received and enacted by participant dyads. Acceptability (i.e., understanding, motivation, sense of achievement) and tolerability (i.e., effort, fatigue) ratings and safety of the intervention were within *a priori* target ranges. Recruitment and retention targets required improvement, due to the COVID-19 pandemic outbreak, as well as the lack of a clear clinical diagnostic pathway for dementia in both sites. Areas for future modification were clearly identified, including: the assessment/delivery logistics circuit; procedures for arranging visits; communication among referring clinicians and the study team.

**Conclusion:** This is the first study in a LMIC of sensory enhancement to improve dementia outcomes. Positive feasibility, acceptability and tolerability findings suggest that a full-scale effectiveness trial, with certain modifications is warranted.

## Introduction

Cognitive decline and dementia are newly emerging as public health priorities in low- and middle-income countries (LMICs) due to aging of the population. In South Asia alone, it is estimated that the number of people who will be living with dementia (PwD) by 2030 will exceed 9 million (1). Approximately one-third of adults over the age of 65 years experiences a disabling hearing loss (2), and in PwD, over 85% are affected (3). Together, cognitive and sensory deterioration can result in a “crucible of co-morbidity” for older people, compounding negative outcomes such as poor quality of life and high caregiver burden (4, 5).

To date, the infrastructure of health and social care services for older people in South Asia is still quite limited (6, 7). However, in contrast to dementia services, hearing services are more developed and there is evidence that improving hearing function in older people represents a potentially reversible cause of cognitive impairment, or, may optimize remaining cognitive and functional ability in people already with dementia (8, 9). Hearing interventions may promote better outcomes for people with cognitive impairment, but consistent evidence for the positive impact is still lacking, highlighting the need for sufficiently powered randomized controlled trials of such interventions on outcomes relevant to people living with dementia (8). As highlighted by recent guidance for up scaling dementia research in Pakistan (10), developing and evaluating low cost, easily accessible interventions for PwD and their families in such low- and middle-income health economies such as South Asia, is essential to support the development of services. Thus, addressing outcomes in dementia by improving hearing is an approach with high potential.

Currently, a large-scale randomized controlled trial (RCT) of a sensory support intervention for PwD is being conducted across clinical sites in five European countries (11, 12), as part of the SENSE-Cog research program (13). The multi-component intervention involves the assessment, management of, and adherence support for hearing and vision deficits in PwD. Early outcomes have indicated that the sensory intervention is pragmatic and feasible (14) and may be effective in improving dementia-related outcomes (15). Thus, SENSE-Cog was deemed a suitable intervention to be evaluated in a South Asian context. However, since the health and care ecosystem in South Asia differs from Europe, and public understanding of dementia and its impact are still developing, it was necessary to undertake an adaptation and feasibility testing program, SENSE-Cog Asia, as a first step, prior to a definitive RCT of effectiveness and implementation.

**Figure 1:**
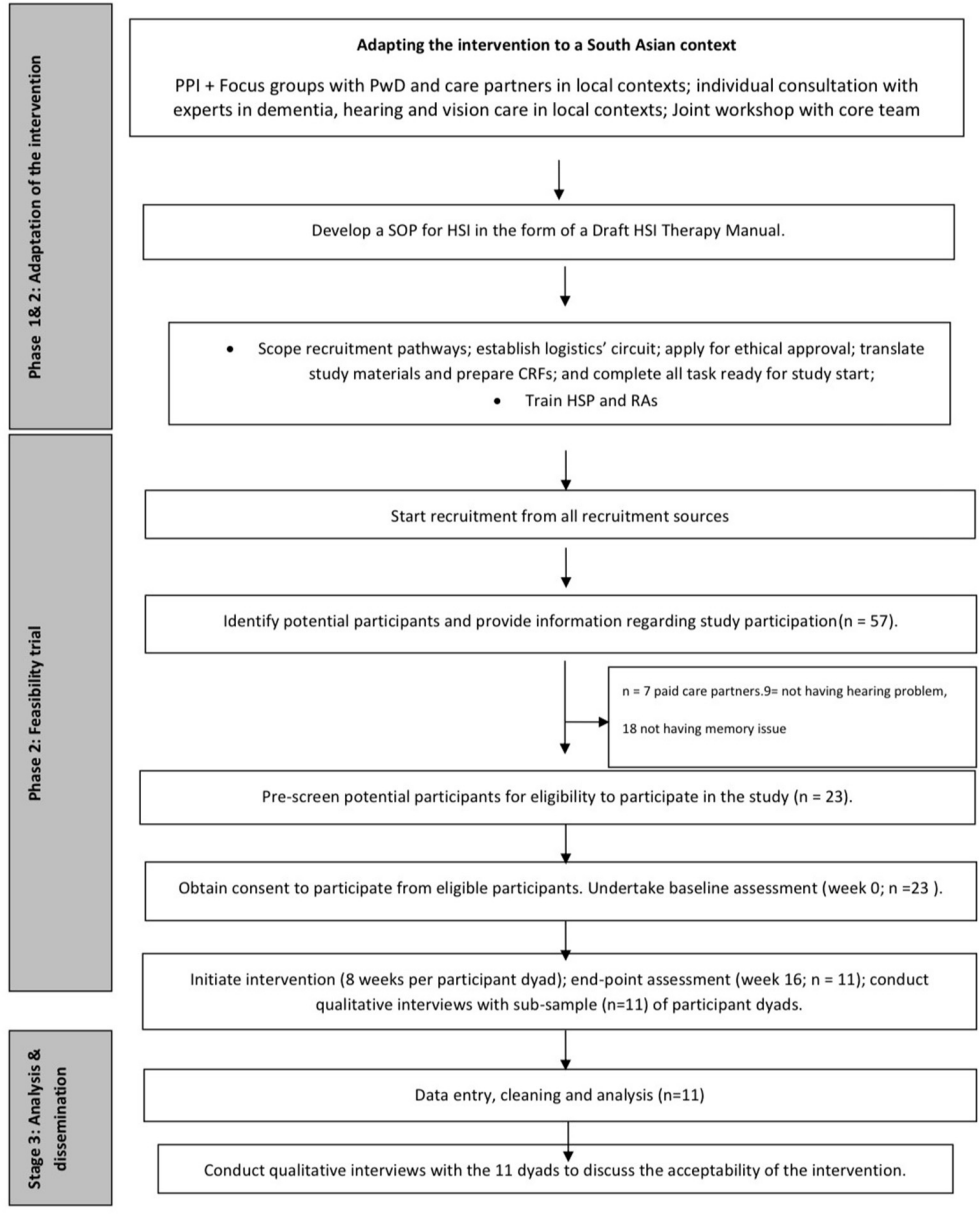
Flowchart of steps in the SENSE-Cog Asia feasibility study.

SENSE-Cog Asia was carried out in three phases, as outlined in Figure 1. Phase 1 was conducted to build capacity and capability for applied dementia research in seven sites across Pakistan (Karachi, Rawalpindi, Lahore), India (Mysuru, Bangalore, Chennai), and Bangladesh (Dhaka). We have reported on this work elsewhere (12). Briefly, to develop an integrated capacity and capability building strategy, we established goals embedded within a Theory of Change framework (16), across six domains: people, research integrity and governance, study delivery skills, international collaborative working, patient and public involvement (including awareness raising, addressing social stigma and health literacy), and development of “pathways” (logistics, referrals, links to existing, or developing services).

Additionally, in Phase 1, we developed a network of patient and public involvement (PPI) groups to inform the work and support adaptation of the intervention. The PPI work, which resulted in a network of people with dementia and their families (*SENSE-Cog Asia Research Advisory Team)*, involved a variety of public engagement activities reflecting different parts of the Wellcome Trust's “Public Engagement Onion” (17). Each site reported PPI outcomes, including changing attitudes and behavior to dementia and research involvement, best methods to inform participants about the dementia study, sharing knowledge and outcomes, and co-adapting the dementia study protocol to the local context. We also reported on the challenges inherent in introducing a PPI model into LMIC settings where hierarchical social structures predominate, particularly in the context of medical professional-patient relationships.

Phase 2 of SENSE-Cog Asia involved cultural adaptation of the SENSE-Cog hearing and vision intervention, which was originally developed (4) and field tested (11) in Europe over 18-months. Adapting an intervention developed elsewhere into a context with a markedly different language, socioeconomic and cultural context requires modification before it can be systematically evaluated (18). This enhances the relevance of the intervention to the local population and increases the likelihood of implementation and “scale-up” following the evaluation stage.

Finally, Phase 3 of SENSE-Cog Asia was initially designed as a feasibility study of the adapted intervention in all seven sites across South Asia. The original sample size was chosen as 70 dyads (PwD and their care partners) (*n* = 7 dyads from each site). The focus of the study was to develop and test the logistics' circuits of the intervention delivery, other feasibility parameters, and tolerability of the intervention by participants. However, following phase 1 and 2, the COVID-19 pandemic broke out and all research activity stopped. The key challenge in recruitment was due to the local lockdown in participating hospital centers in India and Bangladesh due to the pandemic. However, these sites were also slow to open in the first instance due to delays in obtaining approvals from local sponsors and the bureaucracy around transfer of study funds. Only the sites in Pakistan (Karachi and Lahore), were initiated for the feasibility study. Thus, we chose to follow current guidance regarding contingency planning for dementia research in the COVID-19 pandemic (19) and amended our study protocol accordingly to ensure the safety of our research team and participants. This involved halting further start-up activity in the remaining sites, halting recruitment in the Pakistan sites, and completing as many assessments as possible using remote means. We report the outcomes of our amended study, SENSE-Cog Pakistan, here. Ethical approval for the amended protocol was obtained from the University of Manchester's Research Ethics Committee 5 [2019-6061- 9196].

## Objectives

The primary objectives of our amended feasibility study, were: (1) to describe the baseline characteristics of PwD with concurrent hearing impairment, and their caregivers in the Pakistan study sites; (2) to evaluate the feasibility of the operational elements; (3) to evaluate the acceptability and tolerability of the adapted intervention for a definitive trial; and (4) to explore the impact of the intervention on PwD and caregiver outcomes.

### Ethical Approval

Since the study was conducted in collaboration with UK-based investigators, ethical approval was initially obtained from the University of Manchester's Research Ethics Committee (REC) 5 [2019-6061-9707]. Local approvals were also obtained at each site prior to the study commencing. The investigators at each site ensured that the study protocol and all study-related documentation was approved by the appropriate REC, prior to any participant recruitment. All researchers received training in line with the UK's Good Clinical Practice (GCP) guidelines (20).

Following the COVID-19 restrictions, ethical approval for the amended protocol was obtained from the University of Manchester's Research Ethics Committee 5 [2019-6061- 9196]. The outbreak of pandemic resulted not only in halting further start-up activity in the remaining sites, it also halted recruitment in the two active sites, and the remaining assessments were completed remotely as much as possible using remote means and the amended low risk, non-contact intervention was delivered to the participants who had already consented before the outbreak of the pandemic.

The biggest ethical challenge for the study related to inclusion of PwD in a research protocol when they might not have capacity to consent. Unlike in the UK, where the Mental Capacity Act (2005, amended 2018) has specific legislation to safeguard research participants lacking capacity, none of the three countries involved in the original SENSE-Cog Asia protocol has such legislation. Thus, only participants with capacity were included in the study.

### Equitable Partnerships

Since our work involved close collaboration among investigators from multiple regions, ranging from high to very low-income countries, we followed the principles of a “balanced partnership” to ensure equity in our working relationship (10, 21). Power imbalances in international research partnerships can occur, with one partner, usually the partner from the high-income country (HIC), dictating the terms of the collaboration, and the other partner being expected to comply (22–24). This risks the possibility of exploitation and scientific colonialism. In our case, the intervention originated in Europe and was being adapted to South Asian settings, adding to the risk of imbalance. Thus, we strove to ensure equity by: (1) co-developing the study protocol over 6 months via monthly conferences involving all team members; (2) incorporating local solutions for intervention adaptation; (3) ensuring each site's perspectives were included through monthly team meetings; (4) incorporating the feedback from local PPI groups; and (5) sharing of outputs across sites through co-authorship. This approach fostered mutual respect and cooperation amongst team members.

## Methods

### Phase 1: Capacity and Capability Building

The methods for Phase 1 are described elsewhere (12, 17).

### Phase 2: Cultural Adaptation of the Intervention

Our starting point was the parent form of the intervention, the SENSE-Cog Sensory Support Intervention (SSI), which as was developed and field trialed over an 18-month period (4, 8, 11) and is currently being evaluated in a five-nation definitive RCT in Europe (4). For the South Asian context, it was important to consider local factors such as: (1) language and cultural aspects of participants; (2) availability of resources and services; (3) understanding and awareness of dementia amongst individuals and their caregivers; (4) limited recruitment pathways for clinical dementia research; and (5) limited engagement with dementia clinical research amongst local clinicians and PwD and their families. Thus, we modified the intervention for the South Asian context using a stepwise framework for cultural adaptation including information gathering, preliminary adaptation of the study design, and preliminary testing of the modified intervention (25). The final step, “adaptation refinement” will be guided by the findings of the feasibility study we describe here.

The first step of information gathering involved a rapid literature review of existing psychosocial interventions for dementia in LMICs, followed by PPI consultations with PwD and caregivers in local contexts, individual consultations with local dementia and hearing care professionals, and a joint workshop with core team members of the EU SENSE-Cog RCT team and the local research teams. Findings from this work informed initial modifications of each component of the intervention, as well as aspects of the intervention. In contrast to the European intervention, the culturally adapted intervention, or Hearing Support Intervention (HIS) for South Asia included only hearing support, rather than both hearing and vision support. Additionally, all assessment and outcome rating tools were translated into local languages, and the content of the intervention material was modified to include culturally relevant pictures and activities and take account of literary levels of older participants.

Although the Hearing Support Intervention (HSI) that we developed for the SENSE-Cog Asia trial has core resonances with the European SENSE-Cog SSI, outlined by Regan et al. (11), it differed from the European version in several ways including that we did not specify the make and model of hearing aid that participants would receive and we included dementia awareness education for caregivers (Table 1). Moreover, in contrast to the European version of the study, this was as trial targeting a single sensory modality, hearing, only, rather than both hearing and vision. Additionally, the intervention also included dementia education and support for the care partner. This was considered necessary due to the low level of dementia awareness and cognitive health literary of care partners in Pakistan. A complex intervention with multiple parts tailored to the needs of each individual in this context would thus have to include more than just support for hearing impairment. The approach involved these elements being closely aligned and inter-dependent. Finally, our adapted intervention did not include the intervention components relating to social inclusion, referral to support services, as these were limited in the regions in which we worked. We named the intervention provider as a “Hearing Support Practitioner” rather than a “Sensory Support Therapist” to reflect the focus on hearing alone and to ensure that cultural concerns regarding the term “therapist” were avoided.

**Table 1:** Similarities and differences between the SENSE-Cog Europe and Asia interventions.

	**SENSE-Cog Europe intervention**	**SENSE-Cog Asia intervention**
Nature of sensory loss	Hearing and/or vision	Hearing only
Hearing aids provided	Starkey Muse i2400 Mini BTE	Make/model not specified
Intervention components	Provision of hearing aids/glasses Training and support in using hearing aids/glasses Goal setting Communication training Provision of supplementary sensory devices (e.g., lamp) Referral to support services Supporting social inclusion	Provision of hearing aids Training and support in using hearing aids Goal setting Communication training Dementia awareness training for study partner
Intervention provider	Sensory support therapist	Hearing support practitioner
Duration of intervention	Up to 10 home-based visits	Up to 8 home-based visits
Location of intervention	Participant's home	Participant's home

### Phase 3: Feasibility Trial

#### Study Design and Participants

This was a single arm open-label feasibility and acceptability study including participant dyads (PwD and their caregiver) across two sites in Pakistan. Each dyad received the culturally adapted version of the HSI over an 8-week period, in their own homes. All participants were assessed for capacity to provide informed consent to participate in the study, and if deemed to have capacity, provided written informed consent prior to their inclusion. Researchers were carefully trained to undertake capacity assessments in older and potentially vulnerable people. Additionally, all researchers used a checklist to ensure that the key elements of capacity to consent were present and recorded.

##### Inclusion/Exclusion Criteria

As outlined in Table 2, we included people over the age of 60, with capacity to consent, as per the UK's Mental Capacity Act, 2005 (amended 2018). All participants had to be living at home with symptoms meeting criteria for mild-moderate stage dementia due to Alzheimer disease, vascular dementia or “mixed” Alzheimer and vascular dementia. Where a formal diagnosis of dementia was lacking, we included people with evidence of significant cognitive difficulties (using the informant version of the GP-Cog) and meeting diagnostic criteria based on researcher assessment in consultation with clinical expertise from the PI. All had to have a clinically significant uncorrected or partially corrected (e.g., outdated prescription for hearing aids) mild to moderate hearing loss (worse than 35dBHL at 1,000 Hz and above in the better ear). We did not include people with congenital hearing loss. Study partners were informal caregivers in regular contact with the PwD (at least three times per week).

**Table 2:** Summary of participant inclusion and exclusion criteria and characteristics.

**Inclusion**	**Age**	**≥ 60 years**
	Domiciliary status	Living at home
	Cognitive impairment	Diagnosed with dementia as per ICD10 criteria due to the following conditions: Alzhemier's disease (AD) (as per NINCDS-ADRDA) or vascular dementia (VAD), or “Mixed” dementia (AD + VAD) OR evidence of cognitive difficulties significant enough to suggest the presence of dementia without having been formally diagnosed as having dementia, both with and without capacity. This will include those with young or later-onset dementia.
	Stage of cognitive function	Dementia in the mild to moderate stage, as indicated by a Montreal Cognitive Assessment score (MOCA) (26) of scale score ≥ 10;
	Hearing or vision impairment or both	Adult acquired hearing Impairment: defined by a bilateral hearing difficulty, indicated by failure of a pure tune hearing screening test in both ears, defined by hearing worse than 35dBHL at 1,000 Hz and above in the better ear, using the Hear Check device;
	Capacity to consent to the study	Has capacity to provide informed consent to participate in the study defined by the UK's Mental Capacity Act (2005)
	Study partner	is aged ≥ 18 years; and an informal caregiver (where providing care is not the person's primary paid role), such as a significant other of the PwD (e.g., a family member or close friend), who is either co-resident or in regular contact (on at least a weekly basis);
Exclusion criteria	Hearing	Congenital hearing impairments or has complete deafness (profound hearing loss) to prevent them from following study procedures;
	General status	Any unstable, acute physical or mental condition that would preclude participation in the study
		Is currently participating in any other trial of a potentially cognitive enhancing intervention, excluding marketed cognitive enhancing medication (cholinesterase inhibitors);

*MoCA, Montreal Cognitive Assessment (26)*.

##### Recruitment, Screening, and Sample Size

We recruited and screened potential participants from local hospitals (medical and psychiatry outpatient departments) and the community (Alzheimer Pakistan). The agreed sample size in our amended protocol was 21 dyads (PwD and study partner), with seven dyads per site to be recruited over 3 months. This was considered sufficient to evaluate feasibility and tolerability of the intervention and trial protocol. However, due to the outbreak of COVID pandemic, the recruitment was halted at two sites (Karachi and Lahore), and did not begin at one site (Rawalpindi). At the point of study pause, 23 dyads had been screened (*n* = 17 from Karachi, Site A) and (*n* = 6 from Lahore, Site B). Fifteen dyads (65%) met study eligibility criteria and completed baseline assessments (9 dyads from Karachi and six from Lahore). Participant characteristics are outlined in Table 3. Of all PwD participants, the mean age of included participants with dementia was of 66.8 ± 5.7 years and 9 (39.1%) were male.

**Table 3:** Participant demographic and clinical characteristics at baseline (*n* = 15 Dyads).

**Variable**	**Category**	**Participants with dementia**	**Care partner participants**
*n*		15	15
Age (Years)	Mean (SD)	65.2 (5.5)	30.8 (9.9)
	Median (IQR)	64 (8)	29 (10)
	Range	60–80	19–55
Gender	Female	8 (53.3%)	14 (93.3%)
	Male	7 (46.7%)	1 (6.7%)
Duration of cognitive impairment (months)	Median (IQR)	24 (24)	Not Applicable
Duration of memory problems (months)	Range	4–120 Months	
	Mean (SD)	15.53 (2.9)	Not Applicable
Level of cognitive impairment	Median (IQR) Range	15 (5) 10–20	
(MoCA Total score)			
Normal (score ≥26)	*n* (%)	1 (4.3%)	
Dementia sub-type	Alzheimer's disease	1 (6.7%)	Not Applicable
	Vascular	1 (6.7%)	
	Undiagnosed	13 (86.9%)	
Living status of PwD	Living with spouse	9(60%)	Not Applicable
	Living with family	6 (40%)	
Co-resident with participant with dementia	Yes	Not Applicable	14 (93.3%)
Hours per week spent with PwD	Median (IQR)	Not Applicable	168 h (24)
	Range		15–168 h

*MoCA, Montreal Cognitive Assessment (26); SD, Standard Deviation; IQR, Interquartile Range*.

### Feasibility Study Procedures

Procedures are shown in Figure 1. Following informed consent, we screened potential participants for hearing and cognitive impairment using the Sivantos Siemens Hear Check screener (42) and Urdu-version of the Montreal—Cognitive Assessment MoCA (26), followed by caregiver screening. For those who passed screening, a baseline assessment was undertaken, followed by initiation of the HSI, which was then delivered over eight visits by the HSP in participants' own homes. Dyads kept diaries of each visit, and the HSP kept a logbook detailing visits and dyad responses.

#### Evaluation Framework

*A priori*, we established three possible global outcomes for the study, based on a “traffic light” system: (1) proceed to a definitive study; (2) undertake further adaptations and feasibility work; or (3) do not proceed to a definitive study due to lack of feasibility. To ascertain this outcome, we designed an evaluation framework based on a modified version of the ACCEPT framework (43) for feasibility studies, which we have previously used in other studies (44).

Using quantitative measures (Table 4) and qualitative interviews with participant dyads, outcomes were captured at baseline, within 1 week of the last intervention visit, and 4–6 weeks after the last intervention visit (for selected measures only). At each HIS visit, the PwD and their study partner completed diaries with in-house Likert-type scales (e.g., rating each aspect of acceptability and tolerability on a scale of 1 = strongly disagree to 5 = strongly agree) with space for free text. Additionally, the HSP completed a logbook and field notes. We also conducted semi-structured interviews, following a topic guide, with dyads who completed the intervention (*n* = 11 dyads), within 1 week of the final intervention visit. The focus of the interviews was on participants' perception, experiences, and acceptance of the HSI. Eight interviews were conducted in Urdu (national language), and two in Punjabi (local language). All the interviews were transcribed into Urdu and analyzed to retain the essence of the themes. Themes were translated into English for reporting and back translated into Urdu to ensure accuracy.

**Table 4:** Outcome assessment measures.

**Participant with dementia outcomes**		
Quality of life	DEM-QoL (health-related quality of life of people with dementia)	A 28-item questionnaire with options marked on Likert scale from 1 to 4 (a lot/quite a bit/a little/not at all), sum of all items give total score from 28 to 112, where higher score show better quality of life. Item number 1, 3, 5, 6, and 10 were reversed before final scoring (27).
	DEM-QoL-Proxy	A 31-item questionnaire with 4 point Likert scale from “1 to 4” (a lot/quite a bit/a little/not at all). Total of all items give possible score from 31 to 124, higher overall score indicates better quality of life. Item number 1, 4, 6, 8, and 10 were reversed before final scoring. This is answered by carer about PwD (27).
	EQ-5D-5L	This measures five dimensions of health as mobility, self-care, usual activities, pain/discomfort and anxiety/depression) on 5 levels. Five levels of problems (no, slight, moderate, severe, extreme) are marked from 1 to 5 that summed for total score ranging from 5 to 25. Low score indicates no problem in health dimensions. Score measured on Visual Analog Scale (VAS) from 0 to 100, if high indicate the better health state perceived by the patient. EQ-5D-5L-P is a proxy measure (28).
	Short-Form-12 (SF-12)	There are 12 categorical items in this tool. These items measured on liker scale as item 1 from 1 to 5 (excellent, very good, good, fair, and poor), item 2 and 3 from 1 to 3 (limited a lot, limited a little, or not limited at all), item 8 from 1 to 5 (not at all, a little bit, moderately, quite a bit, and extremely), item 9, 10, 11, and 12 from 1 to 6 all of the time, most of the time, a good bit of the time, some of the time, a little of the time, and none of the time). Item 4 to 5 had yes and no options. Two summary scores are reported from the SF-12—a mental component score (MCS-12) and a physical component score (PCS-12). SF-12-P is a proxy measure about PwD answered by caregiver. Higher score indicate better health status (29).
Functional measures	The Hearing Handicap Inventory for the Elderly (HHIE)	A 25 item tool with 3 response categories (“Yes” = 4, “Sometimes” = 2, “No” = 0). Response of all items added to get total score from “0 to 100” that categorized functional impermanent as 0–16: “No functional impermanent,” “17–42: Mild to Moderate” and ≥43: Significant. HHIE–P is a proxy measure answered by carer about PwD (30, 31).
	Activities of daily living in the elderly (IADL-EDR)	An 11 item with 3-response category. Each item was rated for its applicability (yes/no), degree of disability (scored from 0 to 2) and causative impairment (cognitive and/or physical) (32).
	Neuropsychiatry inventory (NPI-12)	A 12 domains tool that assess both severity and frequency of Neuropsychiatric symptoms. Severity ranged as “mild, moderate and severe” while frequency as “occasional, often, several times per week and once or more day.” Each domain is rated on presence and magnitude of symptoms (frequency × severity). The maximum score per domain is 12, with clinically significant symptoms for a given domain occurring at (frequency × severity) scores ≥4. Total NPI scores range from 0 to 144, higher score for both indicate higher severity and more frequency. It is answered by career about participants (33).
**Caregiver outcomes**		
Caregiver-related burden and stress	The Family Care giving Role scale (FCR)	Consists of 16 items on a five-point scale from 1 to 5, which are divided into three sub-scales: (1) satisfaction with the caring role, (2) resentment, and (3) anger. A summative score for the items within each sub-scale is calculated and higher scores indicate higher satisfaction with the caring role and greater feelings of resentment and anger (34).
Knowledge and awareness of dementia	Family Attitude Scale (FAS)	A 30 item tool with 5 point scale from “0 to 4” as “Never, Very rarely, Some days, Most days, and Every day.” Sum of all items score gave the total score. The FAS was associated with the reported anger, anger expression and anxiety of respondents and found higher among caregivers (35).
	Affiliate Stigma Scale	This instrument has 22 items rated on a 4-point Likert scale with three domains (cognitive = 7 items, affect = 7 items, and behavior = 8 items); a higher score indicates a higher level of affiliate stigma (36).
**Participant dyad outcomes**		
Psychological aspects	Patient Health Questionnaire−9 (PHQ-9)	A 9-item tool that record responses from 0 to 3 (not at all to nearly every day). Total score of all items categorized as 0–4 none, 5–9 mild; 10–14 moderate; 15–19 major depression moderately sever and >20 depression severe (37).
	Generalized Anxiety Scale-7 (GAD-7)	This has seven items to assess severity of generalized anxiety disorder. The items are scored on 4-point Likert-scale ranging from 0 “not at all” to 3 “nearly every day.” Scores 5, 10, and 15 signify cut off points for mild, moderate and severe anxiety, respectively (38).
	The De Jong Gierveld 6-item scale loneliness scale	A 6-item scale with 3 statements about emotional loneliness and 3 about social loneliness. Response categories for each item are “Yes/More or less/No.” Negatively worded questions are scored “1” for neutral and positive response while positively worded questions are scored “1” for neutral and negative response. Sum of all items gave possible score range from “0” least lonely to “6” most lonely (39).
Process measures	Satisfaction with Therapy and Therapist Scale-Revised (STTS-R)	A 12-item tool with responses on a Likert scale from “1” strongly disagree to “5” strongly agree. Sum of the all even number items indicates patient's level of Satisfaction With Therapy (ST) and sum of all odd number items score reflects patient's level of Satisfaction With Therapist (SWT); higher the score, greater the level of patient satisfaction. Obtained score may range from 5 to 30 for both categories (40).
	Modified Credibility and Expectancy Questionnaire (MCEQ)	A modified version with six items each with three responses ranges from “1 to 3” (not at all, somewhat, and very). Sum of the score of all items add up to the final score with possible outcome ranging from “6 to 18” (41).

### Feasibility of Trial Procedures

These included recruitment rate, suitability of eligibility criteria, execution of the “logistics circuit” for assessment and supply of hearing aids, feasibility of the participant diaries, data collection methods, suitability of the battery of effectiveness measures, and retention. Effectiveness measures for the PwD included ratings of quality of life, mental well-being, neuropsychiatric symptoms, functional ability (dementia and hearing-related), and relationship satisfaction. Effectiveness measures for the study partner included ratings of well-being, mental health, caregiving-related burden and stress, and relationship satisfaction. Since this was an open-label study, we did not evaluate randomization and blinding procedures.

### Feasibility of the Intervention Components and Implementation

This was assessed by HSP visit completion rates, visit duration and HSP logbook feedback.

### Acceptability of the Intervention

The appropriateness of the delivery and receipt of the intervention was determined by percentage dropouts due to non-acceptability and rate of serious adverse events.

### Tolerability of the Intervention

This was operationalized by percentage dropouts due to intolerance of the intervention and diary ratings of “effort” and “fatigue.” The criterion for “tolerability” was 75% of participants scoring the intervention with the a priori target ranges: ≥ 3/5 for “effort” and “fatigue.”

Semi structured interviews were also conducted with 10 care-partners who attended the hearing intervention sessions with PwD. Interviews were conducted to evaluate and gather evidence for feasibility of the study.

### Data Analysis

#### Quantitative Analysis

As an initial exploration of a novel intervention, our goal was to observe any signal of change across various outcome measures in the dyad. We examined the change between baseline (pre-intervention) and follow-up (post-intervention) by summarizing the distributions of the outcome measures with measures of central tendency (mean or median) and variability (SD or IQR). The small sample size precluded investigation of associations among outcomes. On initial analysis, the covariates of interest were not heavily skewed and mean and medians were similar, thus, we report mean values here.

### Qualitative Analysis

The free text feedback of the participant diaries and SST logbooks, using content analysis (45, 46). The post-intervention semi-structured interviews with the participant dyads, were evaluated using thematic analysis (47) which included familiarization, coding, generating themes, reviewing themes, defining and naming themes. During the familiarization stage, transcripts were read by (ST, AQ), and coding was done to describe the content. Themes were generated by merging codes into a single theme and reviewed among the researchers (ST, MR) to ensure there was accurate representation of the data. Lastly, all themes were defined in order to explain the data. The whole process was supervised by a senior researcher to minimize the bias (NC).

## Results

Details of the feasibility of trial procedures and acceptability and tolerability of the intervention are outlined in Table 5.

**Table 5:** Feasibility of trial procedures.

**Parameter and *a priori* evaluation criteria (if applicable)**	**Findings**	**Evidence to support finding**
**Feasibility of study procedures**		
Eligibility criteria: 15 participant dyad meet study criteria	Criteria are acceptable except: (1) cognitive score cut-offs may be set too high and exclude PwD who may be appropriate; (2) Hear Check screening cut-off may not be stringent enough.	15 of those screened met inclusion criteria^a^. Two participants who screened positive on hearing impairment using the Hear Check were deemed not clinically suitable for hearing aids on full audiological assessment^a^^,^^b^. One participant refused to visit audiologist for full assessment One participant reported an adverse event (not related to study) soon after baseline assessment (before audiologist visit)
Recruitment: • Total target number • Rate	Successful at 11 of 2 sites. Slower than required for a larger trial.	Six at Site A and five at Site B
Retention: ≥10% completed all study procedures	Successful in both sites.	six completed the study in Sites A; five completed in Site B
Screening & baseline process:	Appropriate due to the length of assessment battery.	13 dyads had one visits for screening, 9 dyads had two visits for baseline assessment
Outcome battery administration and suitability:	Outcome rating scales are generally acceptable. Some scales were not suitable for the study population and require revision.	Minimal or no concerns were noted on battery duration and level of difficulty, other than all two sites reporting problems with: Details of the scales are in the table above.
Device logistics circuit:	Broadly feasible; areas for improvement identified.	All participants^a^ received the prescribed hearing aids^b^. Delays in assessment for and receipt of hearing aids affected overall study timelines^a^.
Participant diary: ≥ 70% completion	Diary activity was feasible for both PwD and study partner.	Out of six participants from site A, five completed their participant diary completely and one participant completed the diary till 3rd session only as sessions done on phone due to COVID-19, and participant refused to fill diary^c^ out of five participants from site B, one participant's completed the diary for up to 3 session and two participant's completed the diary over the phone with the research assistant due to COVID^c^
**Feasibility of Intervention components and implementation**		
HSI: Was the his delivered, received and enacted as intended?	It is feasible, although timeline deviations were evident.	10 participants received a hearing assessment within 2 weeks of baseline^a^^,^^b^. One participant's hearing assessment got delayed due to operational issues around audiological assessment 9 participants received their hearing aids within 2–3 weeks and two participants received these within 5 weeks of their audiological assessment. All participants completed intervention component of device skills and knowledge (hearing aids)^b^
**Acceptability of the intervention**		
Was the hearing Intervention appropriate?	The intervention is acceptable	All 11 participants were willing to use their prescribed aids^b^ 0 participant withdrawals due to lack of acceptability
**Tolerability of the intervention by participants**		
HSI:	The intervention is tolerable	All 11 participants were able to complete their hearing assessment^a^^,^^b^. The intervention was completed over a maximum of 12–13 visits

*Key PwD, Person with dementia; SP, Study Partner; HSP, Hearing support practitioner*;

a *Quantiative data*;

b *HSP logbook*;

c *Participant dyad diaries*.

### Feasibility of the Trial Procedures

#### Recruitment and Retention

Over a 6-month period, we enrolled 15 participant dyads across two study sites, giving a rate of 2.8 dyads per month, which was slower than our expected rate of 3.5 dyads per month. Recruitment was slower than expected in Sites A and B, mostly due to the Covid-19 pandemic outbreak, which also prevented Site C from opening. We screened 23 participants dyads across both sites, 15 participant dyads were eligible to be enrolled in the study. Of whom two were excluded following a normal hearing assessment by the audiologist. One participant dyad withdrew consent following the baseline assessments and one participant had an adverse event before the follow-up. At Site A, the reasons for the slow recruitment rate included the retirement of the referring consultant neurologist and a low number of older people with memory complaints attending the local psychiatry outpatient department, which was our main source of recruitment. Additional recruitment was undertaken from community health settings, supported by community workers. At Site B, of the six participant dyads who passed the screening stage, one withdrew due to an unrelated serious adverse event in the PwD after completing the baseline assessment. Reasons for a slower than expected recruitment rate at Site B included: strikes in hospitals, slow approval process for the study from local hospitals, lack of memory clinics and specific services for people with dementia. These factors limited the necessary referral sources. The overall successful screening rate was 65.2%. The retention rate at Site A was 83.3% (one PwD died before second follow-up at site A) and at Site B this was 100%. Screening, baseline and follow up visits were conducted according to the protocol across all sites.

### Suitability of Eligibility Criteria

The audiologist did not prescribe hearing aids to two participants who were enrolled in the study. These two participants screened positive on hearing impairment due to conductive hearing loss and the need for surgery. This suggested that refinements to the simple Hear Check hearing screen were needed. All other inclusion/exclusion criteria were considered as appropriate by investigators.

### Execution of the Service and Device Logistics' Circuit

Audiologist visits after baseline assessment were mostly carried out according to the timeline mentioned in the protocol (i.e., 71% of participants received their hearing assessment within 2 weeks of the assessment). Nine of the participants received their hearing devices within 2–3 weeks soon after audiologist visit. Two participants received their hearing aid within 5 weeks of the audiology visit because of the adjustments which were recommended due to severe hearing loss.

### Usability of Study Materials and Suitability of Effectiveness Battery

All study measures and materials were feasible and acceptable to dyads. A total of 11 dyads completed their screening in one visit and four dyads completed the screening over two visits. Moreover, six dyads completed baseline assessment in one visit and 9 dyads completed this over two visits. There was an impact of COVID- 19 on diary use. Out of the six participants from site A, five completed their participant diary for all sessions while one participant completed the diary for only two session, as sessions were delivered over phone due to COVID-19, and participant refused to fill the diary. Whereas, at Site B, four participants completed their diaries for all the sessions while for one participant, dairy was completed by the researcher as sessions were delivered over phone due to COVID-19 and participant was unable to complete the diary on his own. There were no missing data on the effectiveness outcome measures.

### Feasibility of the Intervention Components and Implementation

We achieved 100% adherence to the study protocol and procedure for HSI at both sites. However, there was an increase in the total number of visits to deliver the intervention mainly due to the outbreak of COVID-19. All dyads completed their intervention over a maximum of 12–13 visits. This included a change from face-to-face to remote delivery of some aspects of the intervention for one dyad at each site due to the pandemic and lock down situation.

### Acceptability and Tolerability of the Intervention

At both sites, there were no dropouts or adverse events due to lack of acceptability of the intervention. Adverse events, which were all unrelated to the intervention, included: death due to heart failure (*n* = 1), fall out of bed (*n* = 1), and hospital admission due to fever (*n* = 1).

### Exploratory Effectiveness Outcomes

Measuring cognition, the most common primary outcome for dementia trials, was deemed not feasible as our study population had varying levels of literacy. Instead, we based our outcomes on a large-scale consultation exercise of meaningfulness of outcomes of non-pharmacological interventions for people living with dementia. This consultation, which involved multiple lay and professional stakeholders, de-prioritized cognitive outcomes and focused more on quality of life and engagement as important outcomes. Furthermore, according to our hypotheses and previous evidence syntheses (8), we did not anticipate that our intervention would significantly impact on cognitive outcomes but would instead have impact non-cognitive outcomes.

### Participants With Dementia

Scores on effectiveness measures at baseline and post-intervention are outlined in Table 6. Overall, improvements were seen in several dementia-related outcomes following the intervention, compared to baseline.

**Table 6:** Baseline and post-intervention outcome measurements for the Participants with Dementia (PwD).

**Outcome domain**	**Baseline** **(*n* = 15)**	**Post-intervention** **(*n* = 11)**	**Post intervention difference**	**2nd Time follow up** **(*n* = 10)**	**2nd Follow up difference**
**Quality of life**					
**DEM-QoL**
*Mean ± SD*	49.9 ± 4.9	65.0 ± 10.0	13.2 ± 9.2	65.3 ± 15.9	13.1 ± 18.2
*Median (IQR)*	49 (5.5)	64 (13)	13 (5.5)	61.5 (13.5)	11 (19)
*(Range)*	(44–61)	(53–84)	(−2 _30)	(41_96)	(−14_48)
**DEM-QOL-proxy**					
*Mean ± SD*	61.7 ± 11.7	78.1 ± 7.9	15.2 ± 17.3	81.1 ± 11.7	14.8 ±17.7
*Median (IQR)*	64 (17)	76 (8)	6 (28)	85 (15)	11.5 (27.5)
*(Range)*	(35–78)	(66–94)	(−3_42)	(58–93)	(−10_39)
**EQ-5D-5L**					
*Mean ± SD*	14.4 ± 3.7	13.5 ± 4.9	−1.7 ± 4.4	Not applicable	
*Median (IQR)*	15 (6)	13 (4)	−3 (1.5)		
*(Range)*	(8–19)	(7–25)	(−5_11)		
**VAS Score**					
*Mean ± SD*	42.7 ± 17.2	47.7 ± 18.5	6.8 ± 24.2		
*Median (IQR)*	45 (20)	50 (12.5)	15 (15)		
*(Range)*	(5–70)	(10–75)	(−55_25)		
**EQ-5D-5L-proxy**					
*Mean ± SD*	13.8 ± 3.7	12.6 ± 4.7	−1.9 ± 3.2	Not applicable	
*Median (IQR)*	15 (5.5)	12 (7)	−3(2.5)		
*(Range)*	(7–19)	(6–22)	(−6_4)		
**VAS score**					
*Mean ± SD*	53.3 ± 19.6	48.2 ± 12.9	−5.5 ± 25.1		
*Median (IQR)*	55 (27.5)	50 (17.5)	5 (45)		
*(Range)*	(20–80)	(25–75)	(−40_30)		
**SF-12 PCS**					
*Mean ± SD*	26.7 ± 4.9	29.5 ± 5.2	3.4 ± 6.1	Not applicable	
*Median (IQR)*	26.4 (5.8)	29.5 (10)	2.7 (8.7)		
*(Range)*	(19.6–37.5)	(23.2–36.2)	(−6.7_13.9)		
**SF-12 MCS**					
*Mean ± SD*	34.0 ± 6.8	40.0 ± 10.2	4.9 ± 9.3	Not applicable	
*Median (IQR)*	33.6 (5.3)	42.9 (14.8)	6.2 (9.5)		
*(Range)*	(25.4–50.4)	(22.3–54.2)	(−12.7_21.5)		
**Functional measures**					
**HHIE**					
*Mean ± SD*	66.7 ± 17.9	11.6 ± 10.5	54.5 ± 21.6	Not applicable	
*Median (IQR)*	70 (30)	8 (8)	56 (24)		
*(Range)*	(36–92)	(00–32)	(4–80)		
**Functional Impairment** *n* (%)					
No	0	9 (82%)			
Mild/moderate	1 (6.7%)	2 (18%)			
Significant	14 (93.3%)	0			
**IADL-EDR**					
CD	9.5 ± 3.0	8.1 ± 3.1	1.0 ±4.8	Not applicable	
	10 (1.5)	9 (2.5)	0 (4)		
	(2–16)	(1–11)	(−9_9)		
PD	2.4 ± 2.6	3.7 ± 3.8	−1.2 ± 3.0		
*Mean ± SD*	2 (4)	3 (7)	0 (1.5)		
*Median (IQR)*	(0–7)	(0–10)	(−9_3)		
*(Range)*					
**NPI-12**					
Total Score	29.3 ± 20.1	9.8 ± 8.8	19.5 ± 11.3	7.7 ± 8.5	21.6 ± 11.6
*Mean ± SD*	22 (21)	8 (18)	14 (25)	8 (13)	16 (24)
*Median (IQR)*	(8–87)	(0–24)	(−5_70)	(0–27)	(−8_87)
*(Range)*					
Delusions	1.7 ± 2.7	00	1.7 ± 2.7	00	1.7 ± 2.7
Hallucinations	0.9 ± 2.1	00	0.9 ± 2.1	00	0.9 ± 2.1
Agitation	2.3 ± 2.6	0.5 ± 0.7	1.7 ± 1.9	0.5 ± 0.7	1.8 ± 1.9
Depression	5.5 ± 3.8	1.8 ± 1.8	3.7 ± 2.0	1.6 ± 1.8	3.9 ± 2.0
Anxiety	4.1 ± 3.3	1.1 ± 1.1	2.9 ± 2.3	0.8 ± 1.0	3.3 ± 2.3
Elation	0.9 ± 2.2	00	0.9 ± 2.2	00	0.9 ± 2.2
Apathy	2.3 ± 2.1	0.7 ± 1.0	1.6 ± 1.1	0.9 ± 1.8	1.4 ± 0.3
Disinhibition	1.5 ± 2.5	0.2 ± 0.6	1.3 ± 2.0	0.2 ± 0.6	1.3 ± 2.0
Irritability	3.4 ± 3.5	0.9± 1.6	2.5 ± 1.8	0.8 ± 1.7	2.6 ± 1.8
Aberrant motor behavior	1.3 ± 2.2	1.2 ± 1.7	0.1 ± 0.4	0.5 ± 1.1	0.7 ± 1.1
Sleep	4.2 ± 4.3	2.7 ± 2.9	1.5 ± 1.4	2.0 ± 2.4	2.2 ± 1.9
Appetite	1.2 ± 1.8	0.6 ± 1.1	0.6 ± 0.7	0.3 ± 0.6	0.9 ± 1.2
*Mean ± SD*					
**Psychological aspects**					
**PHQ-9**					
*Mean ± SD*	17.6 ± 3.8	11.5 ± 3.7	6.0 ± 3.7	12.3 ± 4.7	4.7 ±−5.1
*Median (IQR)*	18 (5.5)	11 (5)	4 (3.5)	11 (5.8)	3.5 (5.8)
*(Range)*	(11–23)	(7–18)	(2–14)	(7-21)	(−2_14)
*n(%)*					
Mild	0	4 (36%)		5 (50%)	
Moderate	3 (20%)	5 (45%)		2 (20%)	
Major	7 (46.7%)	2 (18%)		2 (20%)	
Severe	5 (33.3%)	0		1 (10%)	
**GAD-7**					
*Mean ± SD*	14.2 ± 4.4	6.9 ± 2.9	7.1 ± 3.8	8.4 ± 4.0	4.7 ± 4.5
*Median (IQR)*	15 (5)	6 (4)	7 (6)	9 (6.5)	3.5 (5.5)
*(Range)*	(6–20)	(2–11)	(2–13)	(2–14)	(−1_13)
*n(%)*					
None	0	2 (18%)		2 (20%)	
Mild	3 (20%)	6 (55%)		3 (30%)	
Moderate	2 (13.3%)	3 (27%)		5 (50%)	
Severe	10 (66.7%)	0		0	
**De Jong Gierveld loneliness scale**
*Mean ± SD*	4.9 ± 1.1	4.4 ± 1.8	0.7 ±−1.8	Not applicable	
*Median (IQR)*	5 (2)	5 (1.5)	0 (1.5)		
*(Range)*	(3–6)	(1–6)	(−1_4)		
**Process measures**					
**(STTS-R)**					
Satisfaction therapy	Not applicable	29.2 ± 1.1 30 (1.5) (27–30)		Not applicable	
Satisfaction Therapist		27.7 ± 1.6			
*Mean ± SD*		28 (2)			
*Median (IQR)*		(26–30)			
*(Range)*					
**MCEQ**					
*Mean ± SD* *Median (IQR)* *(Range)*	Not applicable	17.1 ± 1.5 18 (1) (13–18)		Not applicable	

*Dem-QOL(-P), dementia quality of life (-Proxy) (27); EQ-5D-5L, EuroQol Five Dimensions Five Levels (28); VAS, Visual Analog Scale (28); SF-12 (-P), 12 Item Short Form Survey (-Proxy) (29); PCS, physical component score (29); MCS, mental component score (29); HHIE, Hearing Handicap Inventory for the Elderly Screening tool (30, 31); IADL-EDR, Instrument of Activities of Daily living in the elderly (32); NPI-12, Neuropsychiatric Inventory 12 (33); PHQ-9, Patient Health Questionnaire-9 items (37); GAD-7, Generalized Anxiety Scale-7itmes (38); STTS-R, Satisfaction with Therapy and Therapist Scale-Revised (40); MCEQ, Modified Credibility and Expectancy Questionnaire (41)*.

Quality of life as assessed by the DEMQOL [health-related quality of life of people with dementia; (27)], showed improvement of mean score from baseline (49.9 ± 4.9) to first follow-up (65.0 ± 10.0), which was maintained at second follow-up (65.3 ±15.9) 4 weeks later. The DEMQOL-proxy, which assessed quality of life of the PwD as perceived by the caregivers, also showed a 15.2 point improvement in score from baseline (61.7 ± 11.7) to post intervention follow up (78.1 ± 7.9) that was also maintained at second follow up point (81.0 ± 11.09) (see Table 6). Mean health status, as measured by EQ-5D-5L (28), showed improved quality of life at post intervention with 1.7 point reduction in mean score from baseline to post intervention (i.e. from 14.4 ± 3.7 to 13.5 ± 4.9), while the Visual Analog Scale (VAS) score increased from 42.7 ± 17.2 at baseline to 47.7 ± 18.5 at follow up, suggesting improvement in health status. The EQ-5D-5L Caregiver proxy mean scores (28) at the time of baseline indicated average quality of life. Both summary scores from the Short-Form-12 [SF-12, (29)], the physical component score (PCS-12) and the mental component score (MCS-12), showed improvement PCS and MCS mean scores increased from baseline (26.7 ± 4.9 and 34.0 ± 6.8) to post intervention follow up (29.5 ± 5.2 and 40.0 ± 10.2), respectively.

### Functional Measures

Of the 15 participants who completed the baseline measures, 14 (93.3%) had significant impairment and one had mild to moderate impairment on the Hearing Handicap Inventory for the Elderly Screening tool [HHIE; (30, 31)] at baseline, whereas following the intervention, none reported significant impairments on this scale, and two reported moderate impairment, reflecting an overall improvement in hearing-related functional impairment. As shown in Table 6, no changes in instrumental activities of daily living [IADL-EDR scale; (32)] were noted including in the sub-components of the scale. Neuropsychiatric symptom load diminished significantly from pre- to post-intervention (29.3 ± 20.1 to 9.8 ± 8.8) on the Neuropsychiatric Inventory [NPI, (33)]. Additionally, the proportion of behavioral domains which were scored in the “clinically significant” range (≥4 on frequency x severity) following the intervention at follow-up one was significantly lower compared to the proportion at baseline. There was a reduction in mean depression scores on the Patient Health Questionnaire−9 (PHQ-9, 44) from baseline (17.6 ± 3.8) to the first post intervention follow up (11.5 ± 3.7) of more than 6 points on the scale, which is greater than the minimally important clinical difference and was maintained at second follow-up (12.3 ± 4.7). On this same measure, severe depression was found among five (33.3%) participants at baseline that was not present in any of the participant post intervention (Table 6). Generalized Anxiety Scale-7 [GAD-7, (38)] showed a seven-point reduction in anxiety from baseline (14.2 ± 4.4) to post intervention (6.9 ± 2.9); this improvement was also sustained at second follow up (8.4 ± 4.0). Severity of anxiety also reduced from 10 (66.7%) at baseline to minimal anxiety at post intervention follow up (Table 6), as did loneliness scores, a*s* assessed by De Jong Gierveld Loneliness Scale (39).

### Caregiver Outcomes

Caregiver measures at baseline and post-intervention are outlined in Table 7. Overall, as with the participants with dementia, improvements were seen in a number of outcomes following the intervention, compared to baseline. Caregiver burden and stress reduced from baseline to second follow-up, as by an absolute 5.1-point increase in the satisfaction mean score on the Family Caregiving Role scale [FCR; (34)]. Depression and anxiety improved following the intervention, as shown mean score reduced from baseline (9.4 ± 5.9 and 7.2 ± 5.7) to post intervention (7.7 ± 7.0 and 4.5 ± 3.5) and second time follow ups (5.0 ± 2.2 and 3.5 ± 1.9) on the PHQ-9 and the GAD-7, respectively. Severe anxiety was found among two (18.2%) caregivers that reduced significantly at both follow ups. In contrast to loneliness scores among participants in dementia, mean loneliness scores increased among caregivers from baseline to follow up (Table 7).

**Table 7:** Baseline and post-intervention outcome measurements for the caregivers.

**Care partner assessment–self administered**					
**Outcome domain**	**Baseline** **(*****n*** **= 15)**	**Post-intervention** **(*****n*** **= 11)**	**Post intervention difference**	**2nd Time follow up** **(*****n*** **= 10)**	**2nd Follow up difference**
**Caregiver-related burden and stress**					
**FCR**					
*Mean ± SD*	51.5 ± 8.5	52.9 ± 8.8	1.6 ± 9.2	55.9 ± 8.2	5.1 ± 8.2
*Median (IQR)*	50 (9)	51 (4)	0 (10)	54.5 (7.5)	2.5 (7.5)
*(Range)*	(40–68)	(45–77)	(−17_17)	(44–71)	(−5_21)
**Knowledge and awareness of dementia**					
**FAS**					
*Mean ± SD*	38.9 ± 6.8	44.0 ±16.4	−6.9 ± 17.3	Not applicable	
*Median (IQR)*	38 (5.5)	40 (6.5)	−3 (5.5)		
*(Range)*	(25–52)	(33–92)	(−52_14)		
**Attitude and knowledge of dementia**					
*Mean ± SD*	22.0 ± 3.2	34.5 ± 4.6	11.8 ± 4.6	Not applicable	
*Median (IQR)*	23 (3)	35 (8.5)	14 (8)		
*(Range)*	(14–26)	(27–40)	(4–18)		
**Affiliated stigma scale (39)**					
Cognitive	2.5 ± 2.0 3 (3.5) (0–6) 2.9 ± 1.9	12.6 ± 2.0 13 (1.5) (7–14) 12.6 ± 2.2	10.5 ± 3.1 11 (4.5) (4–14) 9.8 ± 2.9	Not applicable	
Affective	3 (1.5) (0–7) 2.5 ± 2.6	14 (2.5) (7–14) 15.1 ± 2.4	10 (4) (5–14) 13.0 ± 2.7		
Behavior * Mean ± SD* *Median (IQR)* *(Range)*	2 (2.5) (0–8)	16 (0) (8–16)	14 (2.5) (8–16)		
**Psychological aspects**					
**PHQ-9**					
*Mean ± SD*	9.4 ± 5.9	7.7 ± 7.0	1.5 ± 7.0	5.0 ± 2.2	4.5 ± 6.0
*Median (IQR)*	8 (9.5)	5 (4)	1 (7.5)	5 (2.5)	3.5 (3.4)
*(Range)*	(2–19)	(2–27)	(−9_14)	(1–8)	(−3_18)
*n(%)*					
None	2 (18.2%)	4 (36.4%)		4 (40%)	
Mild	5 (45.5%)	5 (45.5%)		6 (60%)	
Moderate	2 (18.2%)	1 (9.1%)		00	
Moderately Severe	2 (18.2%)	00		00	
Severe	00	1 (9.1%)		00	
**GAD-7**					
*Mean ± SD*	7.2 ± 5.7	4.5 ± 3.5	3.1 ± 5.0	3.5 ± 1.9	4.3 ± 6.3
*Median (IQR)*	6 (7.5)	3 (2.5)	2 (4)	4 (3)	3 (9)
*(Range)*	(0–17)	(1–14)	(−4_13)	(1–6)	(−4_14)
*n(%)*					
None	3 (27.3%)	7 (63.6%)		5 (50%)	
Mild	5 (45.5%)	3 (27.3%)		5 (50%)	
Moderate	1 (9.1%)	1 (9.1%)		0	
Severe	2 (18.2%)	0		0	
**De Jong Gierveld loneliness scale**					
*Mean ± SD*	3.6 ± 2.1	4.2 ± 1.7	0.6 ± 2.3	Not applicable	
*Median (IQR)*	4 (3.5)	3 (2)	2 (3.5)		
*(Rage)*	(0–6)	(3–8)	(-3–4)		
**Process measures**					
***STTS-R***					
Satisfaction therapy	Not applicable	28.2 ± 2.2 29 (2) (24–30)		Not applicable	
Satisfaction Therapist *Mean ± SD* *Median (IQR)* *(Range)*		27.9 ± 2.3 29 (4) (24–30)			
**MCEQ**					
*Mean± SD* *Median (IQR)* *(Range)*	Not applicable	17.2 ± 1.3 18 (2) (14–18)		Not applicable	

*FCR, The Family Care giving Role scale (34); FAS, Family Attitude Scal (35); PHQ-9, Patient Health Questionnaire-9 items (37); GAD-7, Generalized Anxiety Scale-7itmes (38); STTS-R, Satisfaction with Therapy and Therapist Scale-Revised (40); MCEQ, Modified Credibility and Expectancy Questionnaire (41)*.

Knowledge and awareness of dementia, as reflected by the Family Attitude Scale [FAS; (35)] score among caregivers increased from baseline (37.1 ± 1.9) to post intervention follow up (44.0 ± 16.4), reflecting a possible improvement in attitudes. On the Survey of Attitudes to and Knowledge of Dementia (48) caregivers showed an increase in score from baseline (22.0 ± 3.2) to post intervention follow up (34.5 ± 4.6). Finally, on the Affiliate Stigma Scale (36), initially developed to assess the self-stigma of a caregiver providing care to a family member with a mental illness or intellectual disability and now adapted for dementia, the mean score on all three elements (cognitive, affective and behavior), increased from baseline to post intervention follow up, indicating an increase in caregivers' perceived stigma related to dementia in the person they cared for.

### Process Measures

As shown in Tables 6, 7, high levels of satisfaction with both the intervention and the therapist [Satisfaction with Therapy and Therapist Scale-Revised; (40)] were reported by the participant and caregivers, respectively.

### Qualitative Findings

As shown in Table 8, seven main themes emerged from the thematic analysis of the semi-structured interviews. These were: motivation for participation in the study, views regarding the intervention, impact of the intervention on the participant with dementia and their caregiver, challenges faced due to hearing impairment, understanding of dementia, pathways to care in Pakistan and views regarding the therapist. Table 8 includes exemplar quotes from participants supporting each theme.

**Table 8:** Qualitative findings.

**Main theme**	
**Motivation behind participation** The motivation for most participant dyads to participate in this study was to find a solution of the difficulties associated with hearing and memory impairment. However, due to lack of awareness some participants were initially hesitant to join the study	My mother has hearing impairment and she always gets upset about it. When we try to talk to her, she is unable to hear us. So to make it easier for her we joined the study(PT002) At first I got worried because I was unable to hear what the doctor was saying to me when he came home but she was so nice and dealt with me in such a nice way that made me feel really good later on (PT010)
Reason for participation	
**Views regarding Intervention** Hearing intervention was perceived as acceptable, feasible, easy to understand and useful by PwD. Dyads took interest in all sessions and reported improvement in PWD. There were no adverse event related to the intervention	It felt good when I realized that you have solved my mother's problems by coming to our house and talking to us and giving us hearing aids. It really feels like most of her problems are resolved. It had made us all so happy (PT01). We were not aware that hearing aids can be so useful and they can make the life of the person with hearing impairment so much easier. In fact not only for them, for all of us. Like for any other illness there is a cure and hearing aids are the cure for hearing impairment. It made us feel good and very happy (PT03). One session got delayed because my mother-in-law was not well, otherwise it was all good (PT06).
Acceptability, feasibility & tolerability	After the intervention she was 100% confident in using them and she used them easily (PT04).
**Impact of intervention on PWD** Care-partners coded hearing intervention as effective, in improving PWD's quality of life, mood, social relationships and self-concept. Improved quality of Life	Before getting the hearing aids and the training on how to use them, she used to disturb me all the time even at nights by calling my name very loudly but now she keeps herself busy in different things such as interacting with other people in the family and her friends. She also tries to take care of the loved ones around her. I have even noticed her memory has improved considerably which is making her feel better in herself. The hearing aids you have given are also very advantageous and she is coming back toward life (PT05).
Improved Self Concept	She started to have an inferiority complex, along with negative thoughts all the time considering hearing impairment as a disability but now that its fixed, my mother's mind stays fresh, she is finding it easy to keep herself busy in doing one thing or another around the house (PT01).
Improved social relations	Due to the hearing impairment she started losing interest in social interaction, because it was very difficult for her to talk to someone, but now this device helps a lot and she started interacting with the people again. She also attend different events and gathering in the family now (PT09).
Improved Mood	She used to be very irritated and angry most of the time but now she stays calm. It makes us happy. I also feel fine most of the time now (PT03). Before the death of my father in law, she used to have chickens and their cage but after he passed away, she removed the cage. The therapist advised a cage and me to get few chickens again for my mother in law. I cannot believe how busy she keeps herself to take care of them now. She even help me taking care of my younger child sometimes (PT05).
**Impact of intervention on Care partners** Care-partners also coded hearing intervention as a source for improving/increasing their knowledge, attitude and practices toward dementia. Along with overall impact on their mood, quality of life, and care burden.	The therapist taught us really good communication skills. She advised us to make sure there is enough light so the PwD can see our face when we talk to them. She also advised us to keep PwD company as much as we can so their mind is occupied with the conversation. Yes the therapist gave us really useful tips to deal with the PwD (PT01). I used to think that she was doing everything intentionally but the therapist guided me about her problem and advised me that in this age group, people have this problem but the machine (hearing aid) you gave is very useful.
Improved knowledge, attitude and Practices	I got a lot of relief (PT05). I think I did not have awareness. But now I have a comprehensive guideline that how these things interlink and how to deal with all this (PT10).
Mood	No no, as I explained before that my mind becomes like that, I get irritated sometimes. Otherwise their explaining (I am thankful to God) was very good, there was no problem, and everything was fine (PT01).
Quality of Life	He is much better than before. Both me and my husband were so worried before but now it is much better (PT08). I got a lot of knowledge about how to talk with people who have hearing problem and it has given me confidence that I am able to talk to those people and also help them in day to day life (PT09).
Care Burden	The burden of my father's responsibility is less now. Now he takes care of himself and I can focus more on different chores around the house (PT04). Stress which I used to have 24 h is relieved (PT10).
**Challenges faced due to hearing problem** Hearing problem, highlighted as a main dispute reason between family member and PwD effecting everyone's life with prominent feeling of loneliness in PWD Disputes	Before we had to keep repeating ourselves, and she never really liked anything that I do. Always complaining about me doing everything wrong. I have a small kid and there are so many chores around the house, if she call me repeatedly, how am I supposed to finish my work. She always used to call me when I was doing some important chore and then complaining that I don't respond. This was the main reason of our arguments (PT02).
Loneliness	She used to feel very sad, thinking she is becoming a burden and getting stressed about it. Mostly when we try to talk to her, she prefers to stay quiet (PT01).
**What is Dementia?** Care partners came up with different examples which they were observing in their family member such as forgetting about things, name of the people in the family, not being sure if they had their dinner or not. These were the main symptoms that their brain was getting weak and they are having memory problems. Care-partner's also believed that both memory and hearing problem are interlinked and impacting one another.	My mother in law always seem to forget if she had her dinner or not. When we had guests over, she asked who those people are although it was her own daughter. She doesn't remember when I came even though I stayed with her 15 days. Like did I come on Wednesday or Thursday, she forgets (PT03). Brain becomes weak so they forget where they put things. This causes stress and depression as well (PT06). Stress is the biggest cause (PT01). I think both things were important and may be if there was no device (hearing aid) we wouldn't come to know what is affecting him as he used to become angry when he can't hear or remember something. I think both things are connected (PT10).
Knowledge Regarding Dementia	
**Pathways to care** “Neurosurgeons” and “psychiatrists” were highlighted as person to contact for memory issues. Along with lack of family support as hurdle in accessing this. Pathways	People do things like take them to neurosurgeons or psychiatric hospitals because they have a brain problem, puts things somewhere and forgets. (PT01)
Barriers in Pathways to Care	There is either stress or a lot of house chores, that is why we forget, that is why family members avoid taking them to see a doctor. I am talking about myself (PT09).
**Views regarding therapist** According to the care-partners, sensory support therapist were very cooperative and empathetic. They guided them in such an easier way that it was easy to comprehend and can be understandable to anyone.	The therapist guided us properly and listened to us actively and gave us respect (PT05). She speaks very well, she was very responsible, I mean she empathized our pain (PT08).
Feedback about the therapist	

Regarding participants' understanding of dementia, analysis of the interviews suggested that “brain weakness,” in terms of memory, and emotional distress were considered part of a dementia syndrome. Participants reported that dementia involved memory impairment and impacted on the ability to undertake basic daily tasks. They recognized that help could be sought from “brain doctors,” including psychiatrists; however, participants mentioned barriers in help seeking included the need to undertake household chores, lack of time and encouragement from other family members to take the affected person for appointments, and forgetting to attend appointments.

Caregivers also reported that they gained much knowledge and awareness about dementia and the impact of sensory impairments on the person with dementia's ability to function well. They improved their understanding of hearing aids and felt confident to provide explanations or help others facing same issues. Caregivers also discussed the impact of hearing and memory problem on the mood of the person with dementia and how this fostered family disputes; using hearing aids resulted in improvements in mood and emotional interactions among family members. This echoed the quantitative findings of improved mood, anxiety level and behavioral disturbance seen in the effectiveness outcomes post-intervention.

Participants reported that overall, the intervention was feasible and effective in improving quality of life of the participant dyads. The hearing aids came as a solution to their hearing problem and improved communication, increasing the ability of those with dementia to participate more in daily activities and family interactions. This was reflected in the reduction in loneliness scores in the participants with dementia, as rated on the loneliness scale. Finally, caregivers reported that they appreciated the way the HSP delivered the intervention and felt that the mode of delivery was clear and acceptable.

## Discussion

This is the first reported study of a sensory support intervention for people living with dementia and their caregivers in a LMIC. Non-pharmacological interventions that are accessible, acceptable and affordable, such as the intervention trialed here, have the potential to positively impact the lives of people with dementia and their families, particularly in settings where resources and health literacy for dementia are low. Here, we demonstrated that a home-based hearing and dementia support intervention is feasible, well-tolerated, and acceptable. We also showed that the study procedures were generally feasible, with some modifications, and that the battery of effectiveness measures, were acceptable to participants, had minimal missing data and showed a signal of change pre- and post-intervention. The carefully adapted intervention activities and material were culturally appropriate and received well by the participants. Thus, our findings suggest that a full-scale effectiveness trial, with certain modifications, is achievable, according to our *a priori* “traffic light” criteria. Additionally, this type of study fits well with the applied dementia research agenda in Pakistan (10).

Key areas requiring modification included the need to improve recruitment rates and referral pathways into the study. Finding appropriate services supporting people with dementia proved challenging since the health and care ecosystem for older people's health, particularly for dementia is still developing. Indeed, it was only in 2019 that the country's first official Memory Clinic was opened in the Punjab, in Lahore (verbal communication). As outlined in the “Roadmap for developing dementia research in Pakistan” (10), undertaking applied dementia research alongside service development is essential to ensure the most appropriate, effective and contextually relevant services are put in place. Thus, for dementia research to develop and provide the necessary evidence-base to improve the lives of people with dementia in Pakistan, services and care pathways for dementia need to develop in parallel.

Interestingly, findings from the qualitative interviews revealed that while aspects of participants' understanding of dementia were present, seeking help and support was not prioritized, and barriers such as household chores and “forgetting the appointment” were cited as reasons for not attending clinics. Advances such as the development of a National Dementia Plan in Pakistan (verbal communication, H. Jafri) may help to raise the profile of dementia and support public understanding of the need to seek help and support for a condition that is outside of the normal aging process.

The first phase of the study involved developing the capacity and capability at an individual and team level. This resulted in upskilling new researchers and fostering a research culture in a LMIC-setting with hither to limited experience in older adult clinical research (49). This stage of the work was crucial to prepare the way for a subsequent definitive intervention. Additionally, part of phase one involved recruiting a PPI group in several study sites. This work reported elsewhere (17), was a key element in supporting the cultural and contextual adaptation of the intervention, which was initially developed in Europe for EU settings, which are markedly different to Pakistan and other South Asian settings. The experience of PPI was also unique, since PPI is not well-known nor practiced beyond certain HIC (mostly English-speaking) countries and, moreover, we involved people with dementia and their caregivers. PPI involving in this population is still in its nascent phases, even outside LMIC settings (50).

Another key challenge we faced in our study was the Covid-19 pandemic which arrested clinical research in all settings. We had initially planned to conduct our feasibility study in seven sites in three South Asian countries. However, due to the pandemic, we were only able to conduct the study in two sites in Pakistan, resulting in low numbers of participants and findings from only one South Asian country. We were also required to adapt our protocol to minimize face-to-face assessments. Since few of our older participants had access to online or other remote means of communication, we had to undertake telephone assessments. Despite the challenges, we were able to complete the study according to the amended study protocol, achieve an acceptable retention rate of the participants, and glean meaningful results to inform the next stage of our work.

Aside from our main finding that the study procedures and intervention were feasible and acceptable by people with dementia and their caregivers in Pakistan, we also found that the intervention appears to improve quality of life in people with dementia, and may have a role in improving functional ability and reducing behavioral and psychological symptoms associated with dementia. These findings are in line with Dawes et al. (8) and suggest the need of properly powered control trial of similar hearing intervention. A fully powered sample will also help us further understand the mechanisms of the hearing-cognition relationship (51). Moreover, in caregivers, care burden, distress and depression appeared to improve following the intervention. Results from this feasibility study apply to participants with dementia who have cognitive capacity to provide consent. Feasibility and results for participants with more severe dementia is unknown, particularly as the intervention involves hearing aids (not simpler amplification devices).The outcomes were supported by the qualitative findings from our participant dyads. However, contrary to expectation, affiliate stigma appeared to increase following the intervention, likely due to a greater understanding of the condition of the person being cared for. This suggests that care is needed when increasing awareness and educating family members about dementia as being outside the sphere of normal aging. It is important to mention that while interesting, our findings need to be interpreted with significant caution as the sample size was small, the study was uncontrolled, and the intervention and outcome ratings were not blinded. Nonetheless, finding a signal of change is promising and supports the need to further investigate effectiveness in a fully powered sample, with consideration given to implementation in real life settings.

## Data Availability Statement

The raw data supporting the conclusions of this article will be made available by the authors, without undue reservation.

## Ethics Statement

The studies involving human participants were reviewed and approved by National Bio Ethics Committee of Pakistan (NBC-389) University of Manchester's Research Ethics Committee 5 [2019-6061-9707]. The patients/participants provided their written informed consent to participate in this study.

## Author Contributions

SS, IL, and EH designed the study and developed the protocol, including the intervention adaptation, in collaboration with all members of the SENSE-Cog Asia Working Group. SS the study coordinator. IL project lead, prepared first draft of the manuscript, which was then consulted on by the Pakistan SENSE-Cog team. JM led the PPI involvement in the study. The intervention was delivered by MR and MU. Outcome evaluations were undertaken by SL and RA. ST supervised the qualitative part of the study and analyzed the findings. NZ undertook the quantitative analysis. All authors contributed to the final manuscript.

## Conflict of Interest

The authors declare that the research was conducted in the absence of any commercial or financial relationships that could be construed as a potential conflict of interest.
